# Editorial

**DOI:** 10.1120/jacmp.v9i1.2823

**Published:** 2008-02-11

Although I assumed the mantle of editor‐in‐chief of the *Journal of Applied Clinical Medical Physics* three months ago, I have been serving as the de facto editor‐in‐chief since 1 July 2007. The four and a half months from July to November served as my apprenticeship, as I was learning my new trade under the supervision of Mike Mills. Mike has been a most able and patient mentor during the time it took for me to learn the intricate mechanisms that we use to operate the journal web site, enabling the move from submitted manuscript through review and revision, and through editing, layout, and proofreading eventually to the final result—an e‐published edition of *JACMP*. All who are involved in the journal—editors, reviewers, authors, and readers—owe Mike a tremendous debt of gratitude for bringing *JACMP* to its current situation. When Mike took over as editor‐in‐chief, the journal was in a serious financial condition, and it was not known whether it would survive. Fortunately, Mike took a series of steps that provided *JACMP* with both a viable business model and a viable editorial model, and he left me a journal that is fiscally and editorially healthy, and a pleasure to work on. Thank you, Mike.

Since the announcement that I would take over as editor‐in‐chief of *JACMP*, several people have asked me about the impact factor of the journal, and so I would like to use this editorial opportunity to address that subject.

In combination with number of publications and number of first authorships (among other considerations), the impact factor is often used as a metric to evaluate the productivity of academic faculty members. The impact factor embraces the concept that the journals considered the most prestigious and the most difficult from which to gain a manuscript acceptance have higher impact factors. Publication in a journal with a high impact factor presumably counts more toward academic stature than does publication in a journal with a lower impact factor.

The impact factor of a journal is defined to be the number of times papers published in that journal are cited for a two‐year period after their publication, divided by the number of papers published in that journal during the two‐year period. For example, if *A* is the number of times a paper published in a particular journal in the period 2005–2006 is cited in all journals during 2007, and *B* is the number of papers published in that journal during 2005–2006, then the impact factor of that journal in 2007 is *A* divided by *B*. The mathematics are profoundly simple.

The numbers used in evaluating the impact factor are obtained from an organization called Thompson Scientific, formerly Thompson Institute for Scientific Information. Thompson Scientific is a private organization that, among other activities, monitors activity in the scientific journals. One of its products is evaluation of the impact factor for a very large number of journals.

Now, how does impact factor relate to the *Journal of Applied Clinical Medical Physics*?

Note that the 2007 impact factor is actually released in 2008 and that it relies on publications in a journal from 2005 and 2006. *JACMP* was listed in Thompson Scientific only about two years ago, and so it might be another year or two before an impact factor is published for the journal.

However, the impact factor is not without its detractors. As is the case with many statistics, this particular statistic can be misused, abused, and manipulated. The Wikipedia entry for impact factor (en.wikipedia.org/wiki/Impact_factor) provides a discussion of the pros and cons of its use. The bottom line is that the impact factor may not necessarily be relevant to manuscripts published in *JACMP*.

Why would I say this? Essentially, the impact factor is a measure of citations of a paper. A published paper is cited when another author uses information from that paper in another publication. Consequently, the number of citations of a paper is a measure of the utility of that paper in the propagation of knowledge. But the information presented in our journal is directed more toward improving the clinical practice of medical physics than toward propagating knowledge. Consequently, much of the information provided never finds its way into further publications. The real measure of impact of a paper in the *Journal of Applied Clinical Medical Physics*, then, is the number of times the information in that paper is used by a practitioner of medical physics to improve the quality of medical physics practice.

A better way to assess the impact of clinical journals needs to be identified. Once that better way is found, we will have an effective and relevant metric for evaluating the impact of papers in *JACMP*.

George Starkschall

Editor‐in‐Chief

15 February 2008

